# Exploring high scientific productivity in international co-authorship of a small developing country based on collaboration patterns

**DOI:** 10.1186/s40537-023-00744-1

**Published:** 2023-05-15

**Authors:** Irena Mitrović, Marko Mišić, Jelica Protić

**Affiliations:** School of Electrical Engineering, University of Belgrade, Belgrade, Serbia

**Keywords:** Data mining and analysis, Co-authorship networks analysis, Collaborative behavior, Research evaluation, Scientometrics

## Abstract

The number of published scientific paper grows rapidly each year, totaling more than 2.9 million annually. New methodologies and systems have been developed to analyze scientific production and performance indicators from large quantities of data available from the scientific databases, such as Web of Science or Scopus. In this paper, we analyzed the international scientific production and co-authorship patterns for the most productive authors from Serbia based on the obtained Web of Science dataset in the period 2006–2013. We performed bibliometric and scientometric analyses together with statistical and collaboration network analysis, to reveal the causes of extraordinary publishing performance of some authors. For such authors, we found significant inequality in distribution of papers over journals and countries of co-authors, using Gini coefficient and Lorenz curves. Most of the papers belong to multidisciplinary, interdisciplinary, and the field of applied sciences. We have discovered three specific collaboration patterns that lead to high productivity in international collaboration. First pattern corresponds to mega-authorship papers with hundreds of co-authors gathered in specific research groups. The other two collaboration patterns were found in mathematics and multidisciplinary science, mainly application of graph theory and computational methods in physical chemistry. The former pattern results in a star-shaped collaboration network with mostly individual collaborators. The latter pattern includes multiple actors with high betweenness centrality measure and identified brokerage roles. The results are compared with the later period 2014–2023, where high scientific production has been observed in some other fields, such as biology and food science and technology.

## Introduction

The public attention to extensive bibliometric studies of scientific production has been on the rise in the recent years due to the globalization of science and accessibility of data about overall scientific production through index databases, such as Web of Science (WoS) and Scopus [1]. The papers became readily available to the broader community of researchers, even those from smaller countries with low or modest income, due to the growth of the Internet, the broader coverage of journals, and the trend of open access [2]. Moreover, introduction of the obligatory accreditation process for higher education institutions, and the rising interest for different rankings of world universities, also increased interest in quantitative and qualitative aspects of scientific production.

According to [3, 4], the global scientific production almost tripled from year 2000 to more than 2.9 million publications annually in 2020 [5]. The share of papers co-authored by authors from different countries increased to around 25% in 2016. That all led to difficulties with scientometrics analysis of such large quantities of data. New methodologies [6], workflows [7] and software tools [8] have been proposed to improve information retrieval, storage, and analysis. Just like dynamic business environments, the field of scientific data management imposes all challenges characteristic for the field of big data, often described with “four V” [9], while the methods and means found in big data research gain importance in the field of scientometrics [10, 11]. The *volume* of datasets permanently increases, resulting from intensive globalization of science [12], data *variety* imposes the need for multi-source data integration [13], *veracity* challenges are present due to name disambiguation problems [14], and the speed of data change expressed by *velocity* permanently increases [12].

Even in small developing countries, such as Serbia, with modest GDP per capita of $9230 in the 2021, and investments of less than 1% of GDP into research and development, the increase of number of papers in journals indexed in WoS is noticeable, reaching its local maximum in 2012 with 6485 papers in the article and review WoS category. The period between 2013 and 2018 was noticeable for the small decline and the stable output of around 6100 papers per year, while the new rise started in 2019. In 2022, there were 7121 such papers. The average annual growth of the number of papers by Serbian authors in the SCIe journals during the period between 2006 and 2013 was about 4.8%, similar to the growth rate of about 4.7% per year, estimated in the classical work of Derek J. de Solla Price [15], and in accordance with findings in [4, 16]. The global growth rate was estimated to 4% in the period 2010–2020 [5].

The first broader study of scientific production by researchers from Serbia was presented in [17], focusing on the analysis of the papers that were co-authored only by researchers with affiliations of Serbian institutions. The second study [18] also included into the data set the papers that have participating co-authors from the other countries, analyzing performance measures related to the journal’s distribution, WoS categories distribution, country and institution participation.

The goal of this research was to reveal the overall scope of scientific cooperation and achievements involving prominent Serbian and international authors. We have extracted and processed the papers with affiliations of at least one institution from abroad and at least one national institution from Serbia. We have noticed the presence of “mega-authorship” papers [19, 20] with tens or hundreds, and even thousands of authors in the initial dataset. We further examined the dataset without “mega-authorship” papers and found out several authors with high scientific production and interesting collaboration patterns. We examined those collaboration patterns in the international context using bibliometric and scientometric methods, and collaboration (co-authorship) network analysis.

The main contributions of the paper can be summarized as follows:

- We gathered the dataset with research papers involving at least one author with affiliation from Serbia and one author with affiliation from abroad,
- We identified three interesting collaboration patterns that lead to high productivity in international collaboration,
- We discussed the obtained results and compared them with the findings from the open literature.

## Related work

In this section we present a short survey of published papers related to our research. We discuss the extent of international publishing and its effects to collaboration networks, and scientific performance of individuals.

### International scientific cooperation

There were many attempts to analyze and measure international collaboration [12, 21], which increases research productivity and establishes collaboration networks across countries. The analysis of international collaboration ranges from the co-authorship on individual level, to the wider international collaborations, and internationality of the journals [22–24]. Although the internationality should not be considered as quality per se, the wider national representation may bring the diversity of ideas and introduce benefits for the advancement of science. An analysis performed on 14,000,000 documents indexed in WoS [25] has shown extensive cooperation between developed Western countries, as well as the more collaborative attitude of the high-impact institutions. The distance between collaborating countries also matters and it is explored by emerging discipline called spatial scientometrics [26–28]. It was found that in 2009 10% of single-authored papers had double affiliation. In more recent research [29], the number of authors with double affiliation increased.

Collaboration between countries was studied not only on traditionally trusted bibliometric sources such as WoS or Scopus, but also on the profiles extracted from the Google Scholar Citations [30] and Microsoft Academic Research [31] using dedicated workflows. The conclusions of the study [30] are that the United States dominate the world scientific map. International collaborations over different regions have been subject of many studies during the last decade. Scientific cooperation of the EU countries with other developed regions [32], the trends of international collaboration in Eastern Europe, especially with the EU [33], and the cooperation analysis between sub-Saharan African countries [34] are some of the examples of these studies. Patterns and dynamics based on co-authorship between researchers from Australia and South Korea were analyzed in [35]. The findings showed inverse specializations of the Australian and South Korean researchers with most collaborations being in the field of physical sciences, life sciences, and health sciences, but still with significant participation of the researchers from third countries.

There were also some global studies related to scientific disciplines, such as [36] which included 1.4 million papers in Physics. One of the interesting results is that the first author is rarely from the developing country. Such inequality is also found in more recent study [37]. Social network methods were used in biomedical and clinical research, such as HIV and HPV research [38] and psychiatry research [39]. Collaboration dynamics of Mexican research in chemistry are described in [40]. The results show that more than third of the papers are published with foreign partners, especially those from USA and Spain. Multilateral collaborations were much less frequent. Another study from the social sciences domain in Mexico [41] showed similar results in the context of international collaborations. However, it also showed up that around 42% of the papers were written by single authors, which is a more frequent occurrence in social sciences. A co-authorship analysis of higher education research is presented in [42] with the dominance of Anglophone countries and strong focus on the individual level phenomena, rather than the system- or country based comparisons.

### Collaboration analysis through co-authorship

There are several studies about the impact of co-authorship on the scientist’s productivity and the number of citations. Such studies also include the co-author network analysis that discover the characteristic roles of the individual authors in the co-author community. Specific co-authorship characteristics and patterns depend on the scientific field. The study presented in [43] analyzes the impact of the number of co-authors and highlights the differences in the extent of collaboration by the scientific area, as well as the recent general trend of increasing the number of co-authors in all fields.

In [44], the authors explore social network analysis methods applied to co-authorship networks. They used three centrality measures (degree centrality, closeness centrality, and betweenness centrality) to understand formation of such networks, network position of authors, and their impact on citation counts. Different collaboration characteristics found in mathematics, physics and biology were separately analyzed in [45], using network science methods. Recent study [46] employed both big data analytics and network science methods in large-scale analysis of scientific production of the top 60 countries with the highest number of scientific publications in the world. The results showed prevalence in co-authorship of the western world countries, such as USA, Germany, England, France, and Spain, but also strong ties of US and China.

Hirsch [47] measures the extent of collaboration by the author’s scientific network performance, expressed through a new index determined by analogy with computation of the h-index. The co-authors are ranked according to the number of joint publications, starting with the most prolific one, in order to determine the proposed coefficient in the similar manner as the h-index is calculated, based on the number of citations. Thus, in [48] the focus is on the relevant co-authors instead of the relevant papers of the individual scientist. In the paper [20] the new R coefficient is introduced to measure the level of collaboration of authors.

### Analysis of the Serbian scientific production

Scientific production of Serbia was analyzed in several studies during the last two decades. In [17], they analyzed papers by authors with Serbian affiliations only, while international collaboration was presented in [18]. The effects of the breakup of the former Yugoslavia on scientific links between former Yugoslav republic were investigated in [49]. A comparison of scientific policies, R&D investments, and scientific production of three countries: Serbia. Slovenia and South Korea [50], found that only 0.3% of Serbian population are researchers, which is ten times less than in EU. Serbia invested only 0.5% of GDP in R&D, compared to Slovenia with 1.5%, and South Korea with 3.5% GDP investments.

Citation analysis for researchers from the University of Belgrade was conducted in [51]. The highest citation share of 47% is achieved by 26% of university staff in the area of biomedical research, while 2% of staff in social sciences and humanities had only 1% of overall citation number. The paper [52] describes the electronic library of MISANU, which includes papers from mathematical journals published in Serbia during the period 1932–2011. A detailed analysis was performed on this data, based on the co-authorship network, and it was found that isolated nodes are dominant, as the mathematics is well-known for many single-author papers.

Among various analysis by scientific fields, we have performed an analysis of the Computer Science WoS categories for the period 2006–2013 [53]. It was found that the production in this field was 3.9% of the total Serbian production. The study showed that the share of Serbia was 0.39% in all scientific fields, while in the analyzed data set it was 0.47%. Therefore, it was concluded that the results of computer science disciplines are better than average, mostly due to the interdisciplinary approach.

### Analysis of the scientific production of individual scientists

Theoretical background for the analysis of co-authorship networks at the individual level is provided in [43]. Multiple aspects of cooperation of an individual author are discussed, suggesting to combine bibliometrics with qualitative information about one’s career. The introduction of some new indexes such as $\phi$ index, that combines publication activity with the frequency of joint activity, was proposed, as well as classification of authors. More recent research on citation counts revealed significant inequality, as large proportion of authors’ citations comes from a small percentage of their publications [54]. According to [55] individual performance of an author should be evaluated in the context of the scientific field for a fair comparison.

The paper [56] contains a representative analysis of the co-authorship network of Hannu Oja, an influential statistician from Finland. It was concluded that Oja’s achievements were highly related to his international collaboration. In more recent research [57], the authors studied collaboration patterns of individual researchers during different career stages using ego-centric networks. They identified 13 relative role growth patterns and classified them into four career growth types.

The study [58] has analyzed a large set of co-authorship ego networks based on the information from Google Scholar. The authors found the strong positive correlation between h-index and ego network size. They conclude that high number of collaborations increases information sources, innovativeness, and creativity of the ego, thus affecting its h-index, which further attracts new collaborators. Ego networks were used in [59] to assess the collaborative practices in biomedical, translational research. They found out that translation of scientific knowledge into practical applications greatly depends on the researcher’s interpersonal connections.

The network science approach based on social circle, generalized friendship paradox, and triadic closure theory is adopted in [60] to measure the influence of individual researchers. They showed the positive impact of collaboration on the centrality measures and decrease of average path lengths when number of collaborators increases.

## Datasets and methods

Web of Science Core Collection was used to gather the data for our analysis. Available tools in advanced search were used to refine the results. International collaboration was the main scope of our research in order to complement the work published in [17, 18]. Timespan was set from 2006 to 2013. Beside the complementary dataset, there were two more factors in determination of time boundaries: in 2006 Serbia gained independence after the breakup of Serbia and Montenegro, and in 2013, University of Belgrade was ranked for the first time on the Academic Ranking of World Universities (ARWU) list [61] for Mathematics field, a year later than it appeared on the global list. University of Belgrade is the oldest higher education institution in the country and the only one that was ranked in the top 500 so far.

### Primary and secondary datasets

To gather the primary dataset, we searched for all documents in Web of Science Core Collection SCI-EXPANDED list classified as articles with at least one author from Serbia. To identify Serbian authors, we searched for the authors from “Serbia” for the defined timespan from 2006-01-01 to 2013-12-31. In order to get dataset that includes only international collaboration, we excluded all papers co-authored only by authors from Serbia. Document types included only “Article” or “Review Article”. The search terms are given in the following pseudo-query where *Countries/Regions* field includes all countries except Serbia:

$$\begin{aligned} CU&= Serbia\ and \\ Document\ Types&= (Article\ or\ Review\ Article)\ and \\ Countries/Regions&= (GERMANY\ or\ USA\ or ...)\ and \\ Editions&= WOS.SCI\ and \\ Timespan&= (2006-01-01\ to\ 2013-12-31) \end{aligned}$$

This way, our research focused only on papers with at least one author from Serbia and at least one author from a foreign country. As a side effect, the single-authored papers with double affiliations from Serbia and another country were also included. We consider this anomaly as a consequence of international collaboration resulting from mobility, since those papers connect two institutions from different countries [62].

The primary dataset needed further preprocessing to resolve disambiguation of authors and institutions and produce secondary dataset. Those problems are well-documented in bibliometric literature [63], and several algorithms to solve them were proposed [64–66]. Those techniques were further reviewed in more recent studies [67, 68]. In our context, some of those problems arise from the fact that Serbian language uses two alphabets—Cyrillic and Latin. The Latin alphabet includes letters with diacritical marks: čćžš which are not always correctly transferred to index databases [69]. Other problems are related to the different ordering or spelling of words in personal or institutional names. Institutional names are inconsistently translated from Serbian to English, since in many cases they represent free translation by authors themselves. Some problems are related to typographical errors, change of surname, added names, nicknames, etc.

The significant problem comes from the fact that Serbia changed its name several times during the past three decades due to the breakup of former Yugoslavia. In 2006 the state union of Serbia and Montenegro dissolved in two independent states—Republic of Serbia and Republic of Montenegro. Due to that fact, articles published in 2006 had to be double checked in order to remove papers with authors exclusively from Montenegro. To parse the primary dataset, we developed an in-house software solution in Java. The main goal of the software solution is to parse the imported full WoS records retrieved in MS Excel format. The output dataset contains one record for each author and publication. The parser is connected with the database which is used to resolve the identity of the authors by taking into account their affiliation. The parser uses Apache POI library for data processing and it stores data in PostgreSQL database. After processing of the primary dataset, secondary dataset is used for further analysis. The source code is available at the following link: https://github.com/orionsam/afi_parser.

### Collaboration network construction and analysis

Collaboration network for individual authors is constructed by deriving data from secondary data set described in previous section. Secondary data set can be represented with undirected, weighted graph $G(V, E)$, where $V$ presents nodes (vertices), and $E$ presents edges (links, ties) between nodes in the graph. Authors (actors) represent nodes, while edges represent relations between them. If two authors co-authored at least one paper together, then an edge exists between them in the graph. Relations are symmetric. Edge weights present the number of times two authors co-authored a paper together.

To analyze scientific collaborations of a scientist (author), we need to derive a subset of nodes from the whole, “sociocentric” collaboration network. This collaboration network contains the principal author (ego) and his scientific collaborators (alters), and edges between them. An edge exists between ego node and all his alters, but also between the alters that co-authored a joint paper with ego. Networks built around ego node are called egocentric networks. To characterize the collaboration networks in this analysis, we used ego network measures described in [70] which are based on “sociocentric” measures used in network science, such as centrality measures, clustering analysis, brokerage, and similar. We used NodeXL [71] and UCINET [72] tools for data visualization and analysis.

## Identifying the most productive scientists

To identify the most productive scientists, we analyzed our primary dataset based on Web of Science data. We identified all authors in the dataset and sorted them by the number of papers they authored in the data set. The top 20 authors by the number of papers are shown in Table 1. The results were surprising, as the top of the list was occupied exclusively by non-Serbian scientists.

**Table 1:** Top 20 most productive authors in the primary dataset

Author	No. of papers	Author	No. of papers
Wang J	555	Liu H	508
Masetti L	554	Rose A	495
Bocci A	554	Gauthier L	494
Zhang J	553	Bai Y	488
Banerjee S	552	Weber M	462
Yang Y	549	Shrestha S	432
Kim H	549	Wagner P	416
Xie S	543	Kim MS	393
Giunta M	512	Chang P	369
Zhang Z	510	Collard C	361

**Fig. 1:**
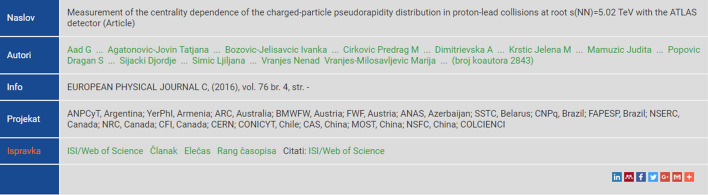
A multi-authored paper with 2843 co-authors, as displayed in national KOBSON system

Identification of the most productive scientists from the national scientific community was to some extent skewed by the existence of research papers with hundreds of authors. Those papers mostly come from the domain of physics, biomedical and clinical research [20]. The number of those papers have been increased in the past decades, with the first paper having more than 1000 authors that appeared in 2004, and the paper with 3000 authors appearing in 2008 [19]. The problem still attracts interest from scientific community in the more recent years, as it raises many ethical problems [73]. Most of these papers come from several well-known institutions, such as CERN and its Large Hadron Collider research group, ATLAS group, CMS group, etc.. An example of such paper from Serbian national KOBSON system is shown in Fig. 1. For such papers, the system displays the first author, Serbian authors, and total number of authors.

We identified 556 of those papers in our primary dataset with at least one co-author from Serbia before disambiguation resolution with our software tool. The list is topped with the names of scientists obviously not from Serbia, as their affiliations are from foreign institutions. The usefulness of bibliometric analysis of such list is questionable, since statistics of authors, institutions and countries show unexpected results, while share of Serbian coauthors expressed by fractional analysis, is minor. The same problem is observed in similar contexts for other countries, such as India [74]. However, it is certainly one way to increase international collaboration through specific collaboration pattern, which is not individual, but institutional in its essence and caused by international mobility of scientists.

For all reasons, we decided to exclude multi-authored papers with more than 50 authors from further research, due to the different collaboration pattern of co-authorship in such papers. Exclusion is performed with Web of Science advanced search tools. The similar approach was used in [17] where bibliometric analysis of papers authored by solely Serbian scientists was performed.

After the exclusion step, we produced the final list of the most productive national scientists in the dataset. Top 10 authors by the number of papers are shown in Table 2. The list is topped by Gutman I with 152 papers, and Stevic S with 111 papers in the analyzed period. Further analysis with our software tools has shown that due to affiliation problems Petrovic S, Pavlovic M, Jovanovic D, Stevanovic D are not unique identities. The numbers of papers next to those names present the sum of all papers authored by authors with those identities. Taking all that into account, it was obvious that the most productive Serbian authors in international collaborations for time period 2006–2013 are Ivan Gutman and Stevo Stević. Both authors have multiple affiliations, one as Serbian scientists, and another as scientists from Saudi Arabia. Our findings were consistent with those found in open literature [17] and newspaper reports [75] which confirm those two scientists as most productive Serbian authors. Before exclusion of multi-authored papers the top Serbian author was Bozovic-Jelisavcic, ranked 64.

**Table 2:** Top 20 most productive authors in the primary dataset

Author	No. of papers	Author	No. of papers
Gutman I	152	Romcevic N	57
Stevic S	111	Petrovic ZL	58
Petrovic S	70	Kaluderovic GN	57
Pavlovic M	69	Mitric M	56
Jovanovic D	60	Stevanovic D	55

Ivan Gutman was a full professor of physical chemistry at the University of Kragujevac, Faculty of Science, and he is a full member of Serbian Academy of Sciences and Arts (SASA). He retired in 2012, and became a professor emeritus at the same university. According to his personal biography [76], professor Gutman holds two Ph.D. degrees, in theoretical organic chemistry (1973) from the University of Zagreb, and in mathematics, from the University of Belgrade (1981). He is a member of several international science academies, and he stayed abroad as research fellow or visiting professor on numerous occasions. Ivan Gutman has been a member of a number of editorial boards, and holds editor-in-chief position in MATCH: Communications in Mathematical and in Computer Chemistry, journal listed on WoS JCR list. MATCH journal belongs to three Web of Science categories: Chemistry, Multidisciplinary, Computer science, Interdisciplinary applications, Mathematics, Interdisciplinary applications. Since 1971, his scientific production includes more than 1490 papers with more than 40,000 citations according to his personal biography, and several books.

Stevo Stević is a full research professor at Mathematical Institute of the SASA. He obtained Ph.D. degree from the University of Belgrade in 2001. According to various reports from Science Watch, Stevo Stević was awarded Rising Star award [77] four times for the largest citation increase (July 2008, November 2008, January 2009, May 2009). He was ranked on the list of the world’s most influential scientific minds in the domain of mathematics in 2014, and was awarded Highly Cited Researcher in the field of Mathematics in the period 2014–2018. Up to date, he authored more than 426 scientific papers indexed by Web of Science with over 13,100 citations.

## Individual collaboration analysis

In this section we analyze scientific production of the two most productive authors, based on the secondary dataset that we have defined earlier. In order to analyze different aspects of their international collaboration, we first performed statistical analysis, and then we analyzed their own collaboration networks with social network analysis metrics. Our aim was to show similarities and dissimilarities that led to publishing performance significantly higher than other scientists.

### Statistical analysis

Statistical analysis is used in our research to assess the scientific production and collaboration patterns of those two authors with quantitative approach. In order to reveal the distribution of papers in secondary dataset over authors, countries, WoS categories, journals, number of co-authors per paper, and type of authorship (first author, corresponding author, other), we have used two methods: normal counting and fractional counting.

In normal counting, each paper is counted as one, independently of the number of authors. In fractional counting [78, 79], each paper is counted as a fraction $1/N$, where $N$ is the number of co-authors of the paper. Moreover, we used fractional counting for distribution of papers per country and distribution of papers per WoS categories. In the case of the former, the credit for the paper is distributed equally to the co-authors, and then accumulated to the countries listed in their affiliations. In the latter case, the paper is assigned equally to all categories that the publishing journal belongs to.

### Number of authors per paper

**Fig. 2:**
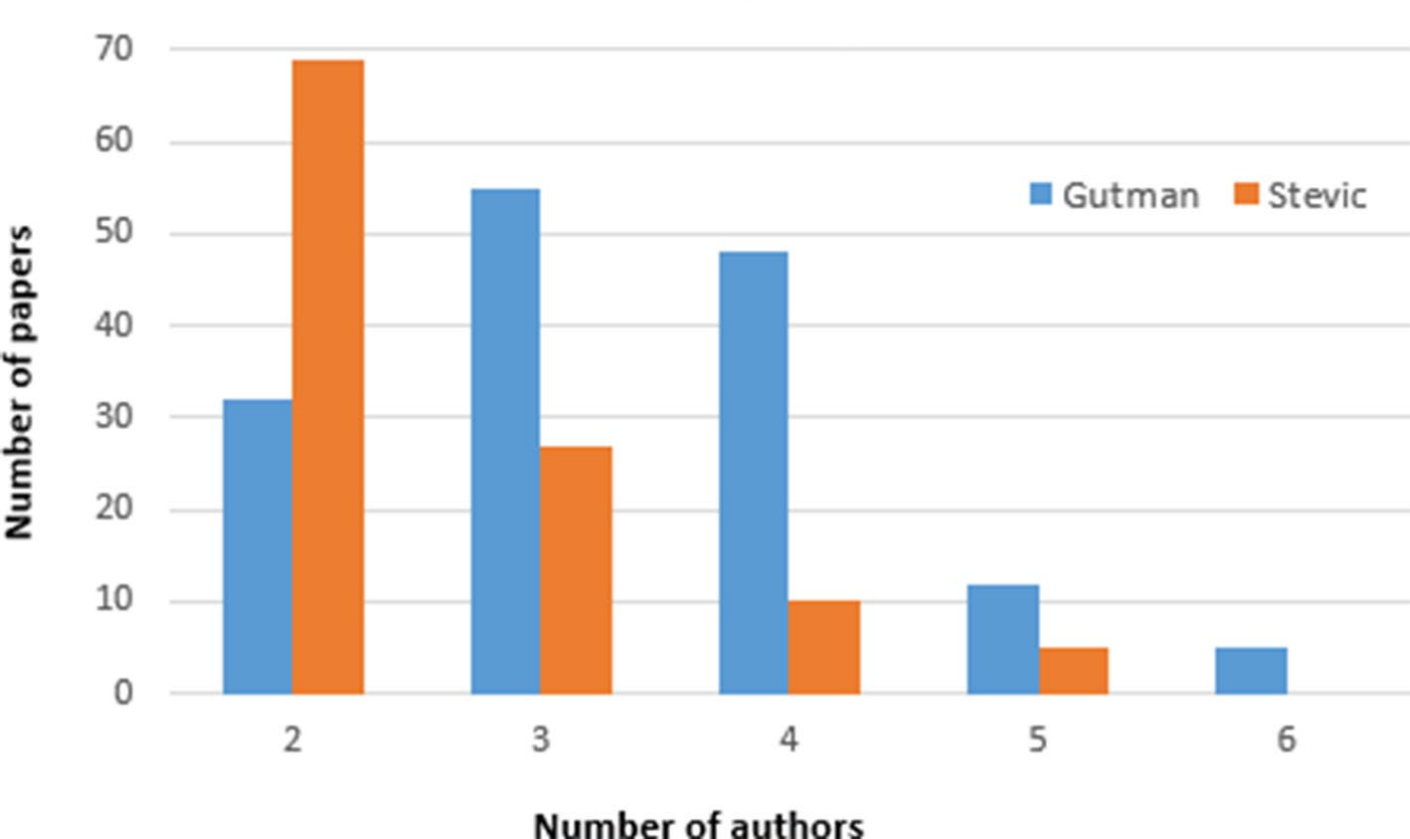
Distribution of papers per number of authors

In this analysis, we consider the number of authors per paper, average number of authors per paper, and distribution of papers per number of authors, in order to study collaboration pattern of an author. The distribution of papers per number of authors is shown in Fig. 2. Ivan Gutman co-authored papers with maximum of 6 authors, while Stevo Stević reached the maximum of 5 authors. Distribution of papers per number of authors shows different patterns for those two authors. The number of papers obviously decreases with the number of authors for Stević, while for Gutman, most of the papers were co-authored by three or four scientists. Both Gutman and Stević can be considered as rapid growth authors, according to [57].

The average number of authors per paper differs for Ivan Gutman (3.36) and Stevo Stević (2.56). The number of authors per paper varies in different scientific fields. Several studies, such as [62, 52], show that mathematicians tend to write papers with smaller number of authors. In [52], the authors investigated scientific production of Serbian mathematicians, while in [62] the same conclusion was derived for the years 1980, 1990, and 2000 in the world production. This trend has roots in the past, since papers in the field of mathematics were almost exclusively single-authored five decades ago [62]. Since Stević is a mathematician his distribution of papers per number of co-authors corresponds with those trends. On the other hand, Gutman is involved in research in several scientific fields, with an emphasis on physical chemistry. According to [62] the average number of co-authors in the field of chemistry was increased from 2.7 to 3.6 from 1980 to 1998. In that sense, our findings correspond to trends in global science.

### International collaboration per country

**Fig. 3:**
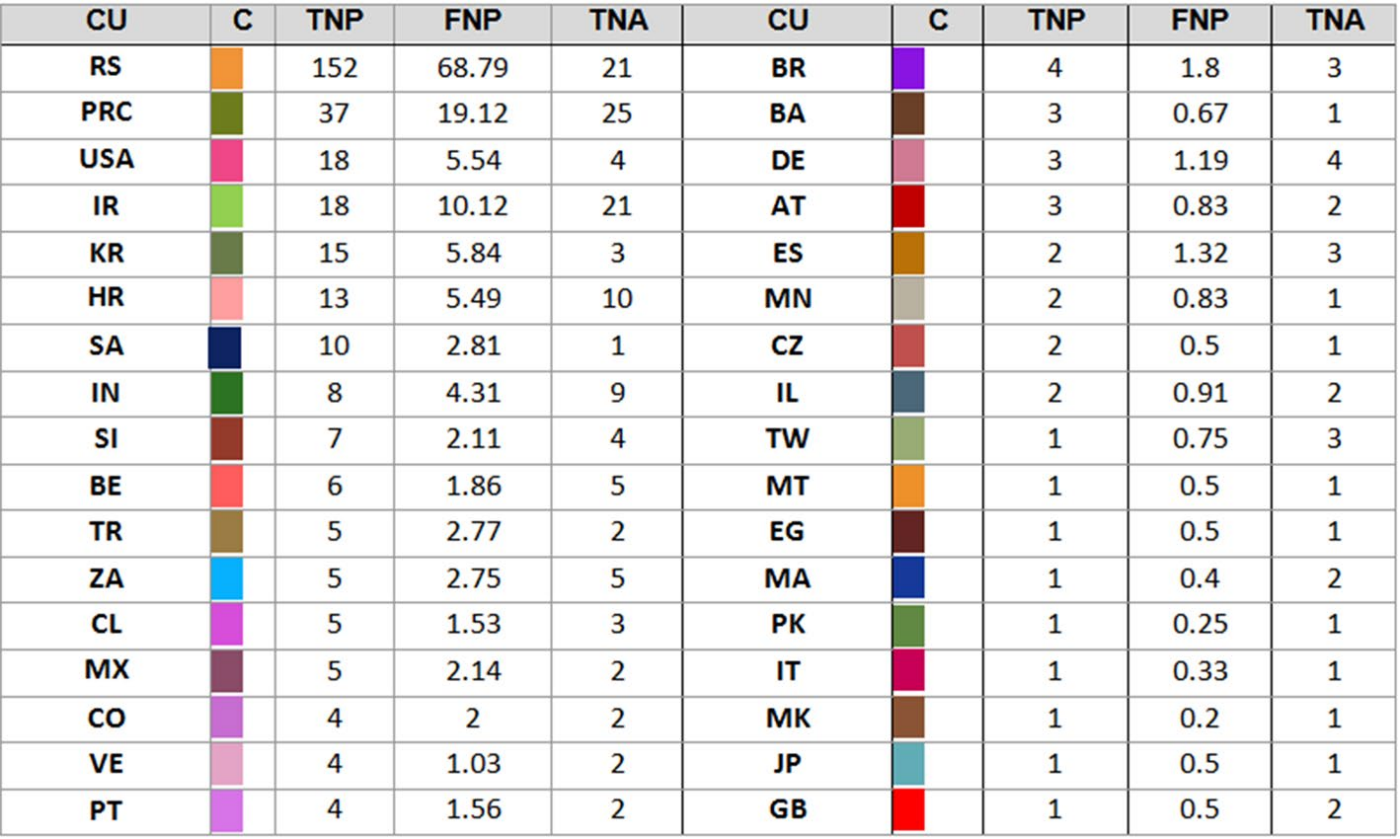
Total and fractional distribution of papers per country for Ivan Gutman; the color in the second column corresponds to the color of the nodes in the collaboration graph (CU: country; C: color; TNP: total number of papers; FNP: fractional number of papers; TNA: total number of authors)

**Fig. 4:**
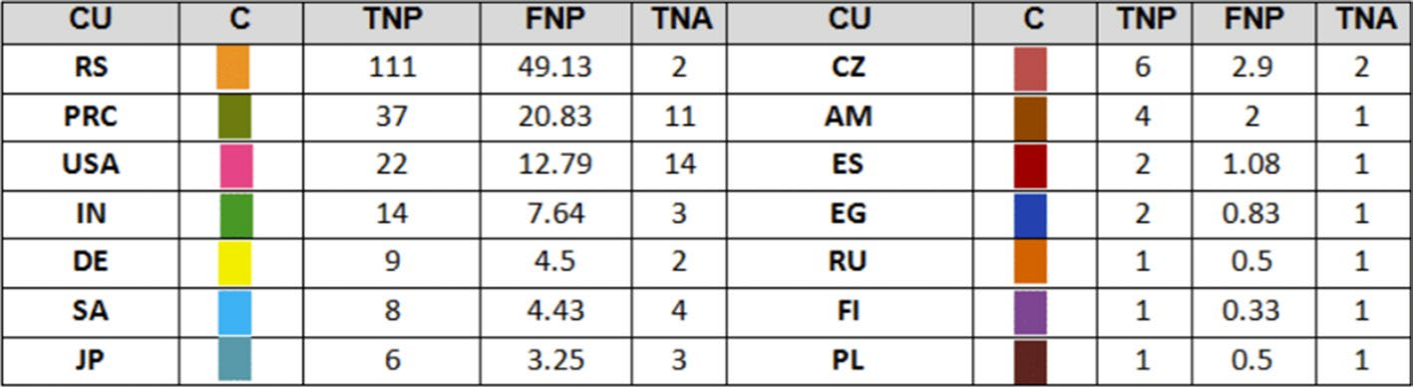
Total and fractional distribution of papers per country for Stevo Stević; the color in the second column corresponds to the color of the nodes in the collaboration graph (CU: country; C: color; TNP: total number of papers; FNP: fractional number of papers; TNA: total number of authors)

Figures 3 and 4 present total and fractional distribution of papers over collaborating countries, for Ivan Gutman and Stevo Stević, respectively. In both tables, column C contains a color that is used to draw the nodes and edges in the collaboration graphs.

Ivan Gutman has broader scope of international collaboration which involves 33 countries, while Stevo Stević collaborators come from only 13 countries. This difference probably comes from two facts. First, as mentioned in the previous section, articles published by Stevo Stević have less co-authors on average than articles of Ivan Gutman. Second, Ivan Gutman has longer lasting research career, which started in former Yugoslavia, in early seventies. For that reason, some of Ivan Gutman’s collaborators come from the countries that emerged after the breakup of Yugoslavia. For Stević that is not the case because no one of his co-authors come from the ex-Yugoslavia republics.

Both authors have intensive cooperation with China and the USA, as shown in Figs. 3 and 4 which is in compliance with presented related works analyzing international collaboration. If we exclude ex-Yugoslavia countries, both authors do not have intense collaboration with other European countries. Fractional counting did not show larger discrepancies in ranking of the countries. The only notable exception is Iran, which becomes the second on the list for Ivan Gutman. According to [46], most of the countries both Gutman and Stević collaborated with are categorized in the first cluster which comprises of developing countries, such as Eastern European and Islamic countries, but also Spanish, French, and Portuguese-speaking countries.

**Fig. 5:**
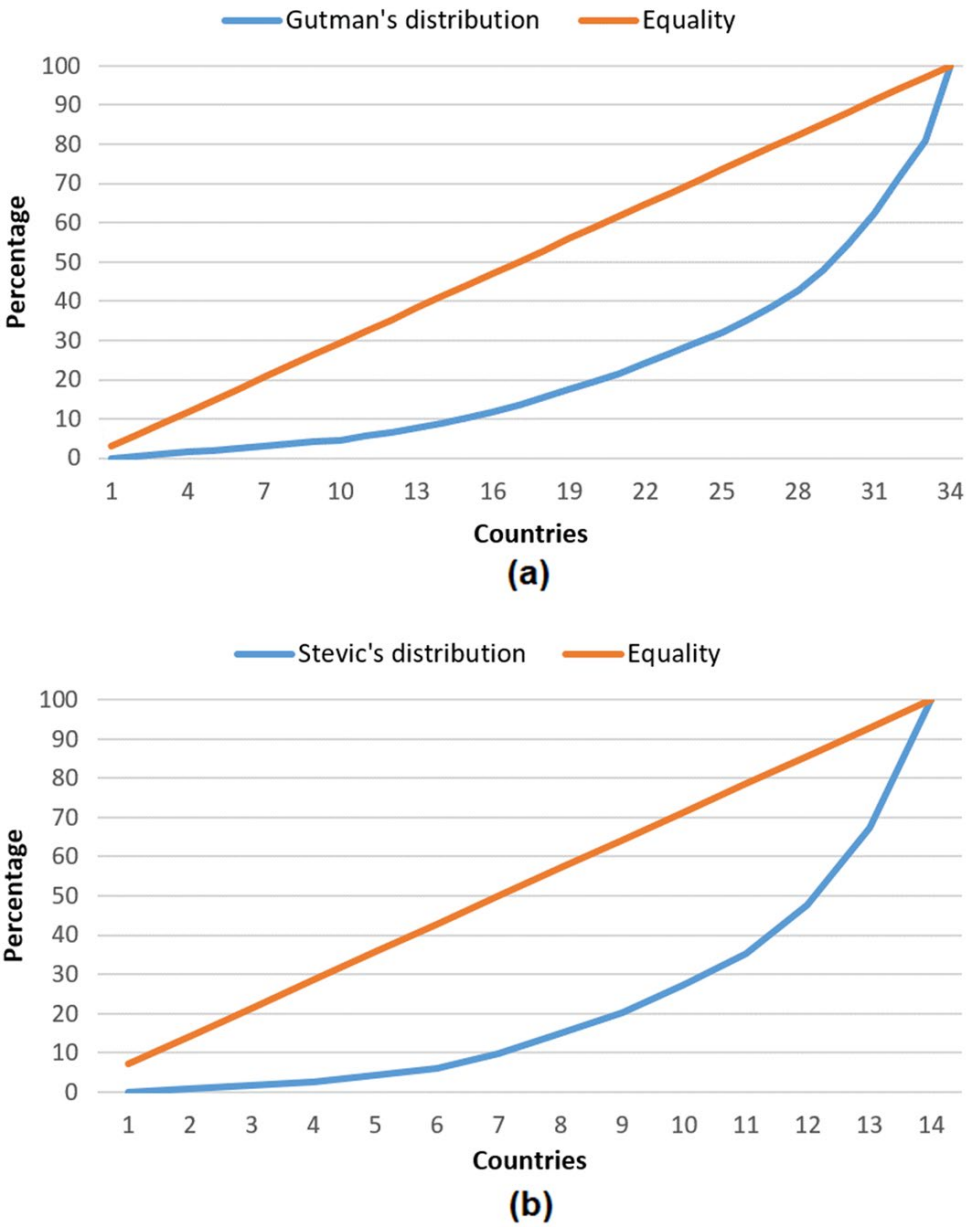
The Lorenz curve maps the cumulative papers share on the vertical axis against the number of countries on the horizontal axis; **a** Lorenz curve for Ivan Gutman; **b** Lorenz curve for Stevo Stević

In order to describe inequality of cooperation with different countries, we calculated Gini coefficient and drew Lorenz curves, using the methodology similar to [36]. Lorenz curves are shown in Fig. 5. Gini coefficients for both authors are similar: Gutman (0.60), and Stević (0.62), which represent high level of inequality for both authors as expected. Although the number of countries span is wider for Gutman, Lorenz curves show similar cumulative inequality trends for both authors.

### Types of authorship per paper

**Fig. 6:**
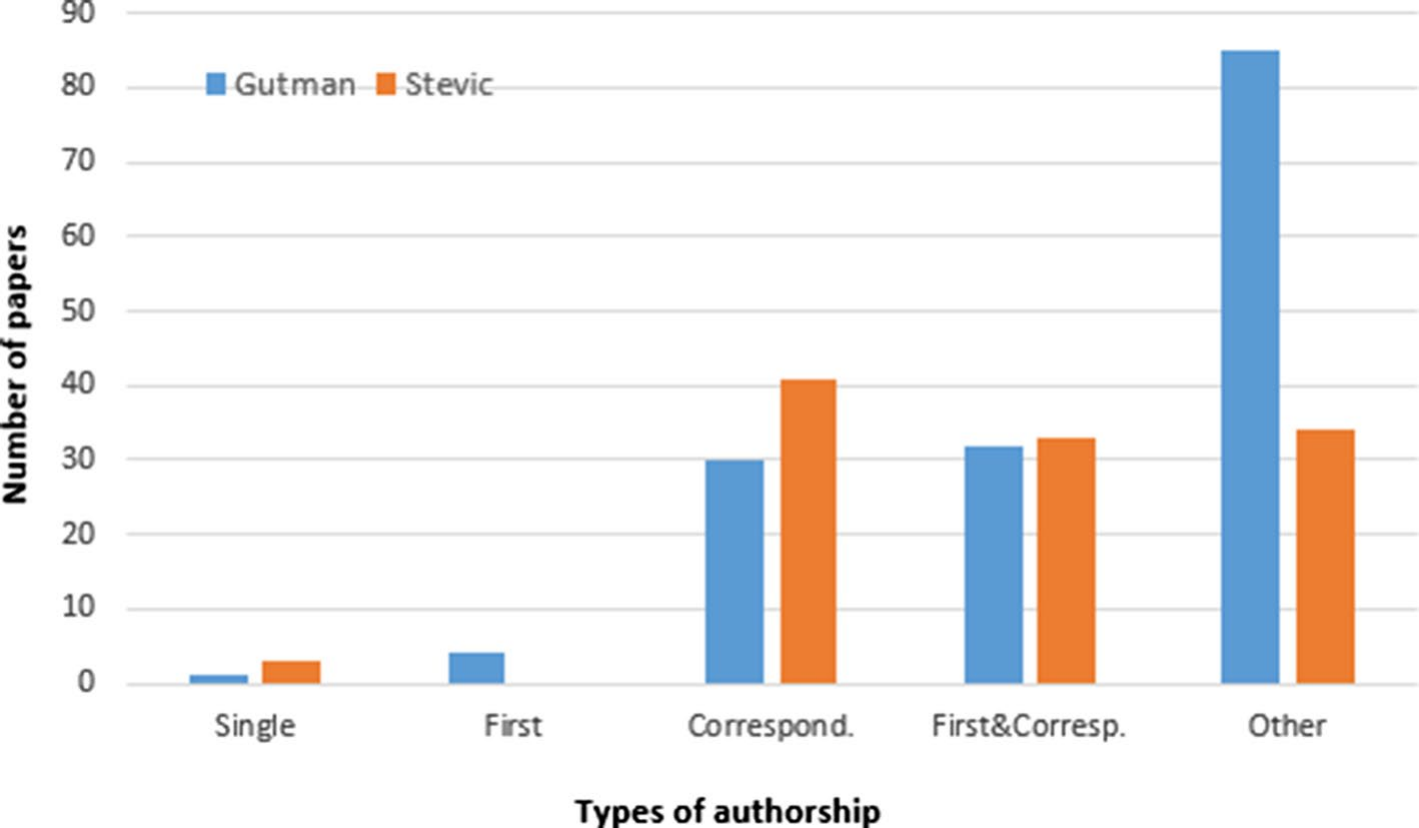
Distribution of papers by the type of authorship

Evaluating procedures sometimes take into consideration the author’s position in the affiliation list. In our analysis, the types of authorship are: first author, corresponding author, first and corresponding author, single author, and other. The corresponding author is referred as reprint author in WoS. The analysis is presented in Fig. 6 and shows that Stević is first, corresponding, or first and corresponding author in two thirds of his articles. On the other hand, Gutman is affiliated in more than half of his papers as an author only.

Different studies, such as [48, 79], consider different author credit-assignment schemas, and discuss the author’s position in the affiliation list. Some evaluation metrics, such as Y index [17, 80], also take into account authorship type (first and reprint author). Y-index consists of two parameters ($J, \theta )$, which assess both the publication quantity, and character of contribution as a single index. In [80], it is defined with the following equations:

1 $$J = \sqrt{FP^2+RP^2}$$

2 $$\theta = atan(RP/FP)$$

FP is the number of papers with the role of the first author, while RP is the number of papers with the role of the reprint author. We calculated Y index for both authors: Ivan Gutman (73.06, 0.96) and Stevo Stević (85, 0.88) using our secondary dataset, which is complementary to the dataset used in [17]. Both authors have more articles in which they were corresponding authors, than articles for which they were first authors. That is in accordance with findings of aforementioned studies, while the quantity is not comparable due to different datasets.

### Journal analysis

**Fig. 7:**
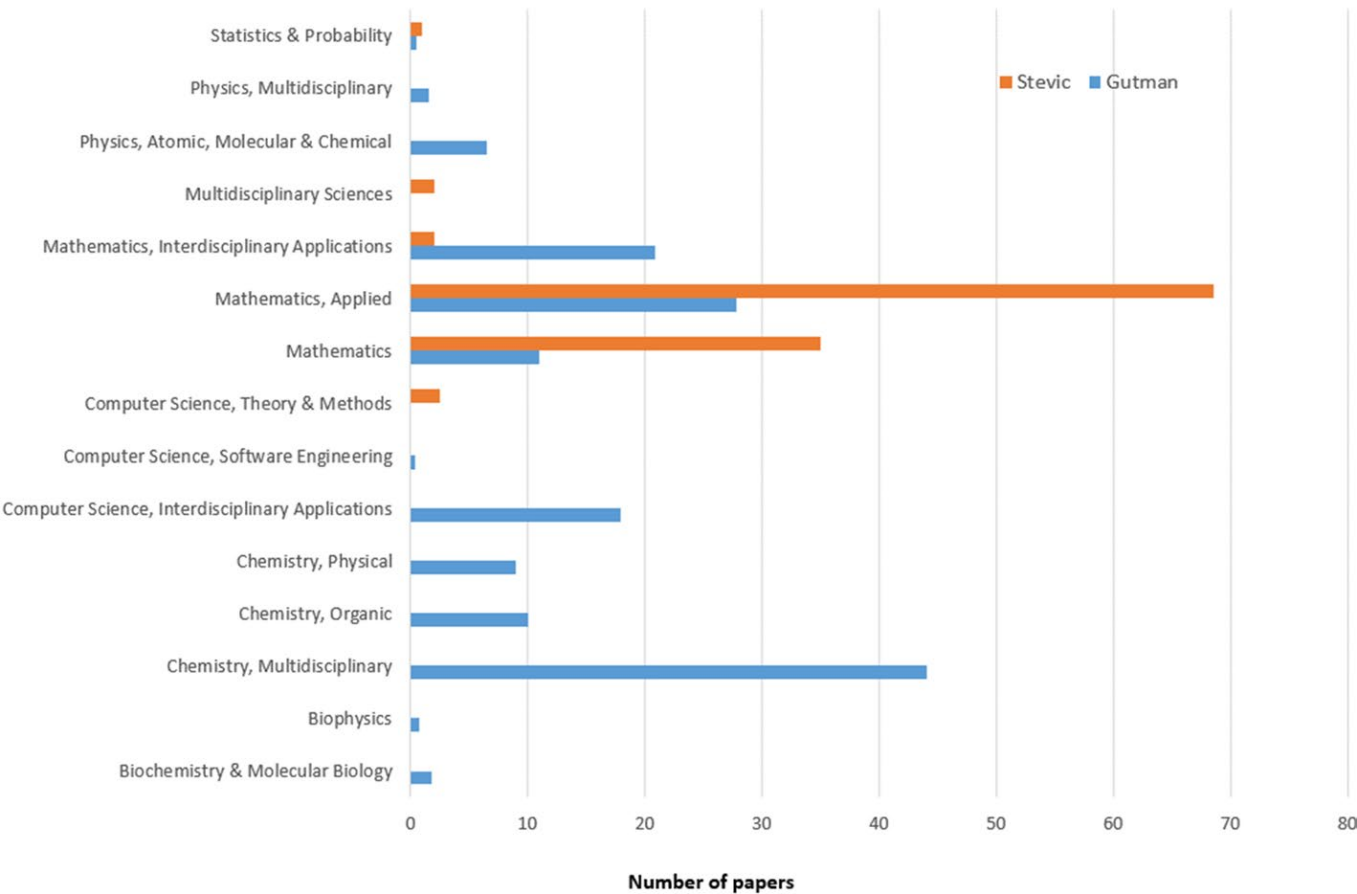
Distribution of papers by journal WoS category

In order to better identify the particular journals and therefore the research subfields of the scientific production by Gutman and Stević, we have analyzed the categories of journals that published their papers. Although there are multiple classifications of journals per research fields, we have used WoS categories, since our dataset was extracted from the same resource. Fractional counting was used, since some journals belong to multiple WoS categories. The equal weight was assigned to each category that the journal belongs to. Distribution of papers by journal WoS category is presented in Fig. 7.

The analysis shows that Stević predominantly published papers in journals from the categories Mathematics, Applied and Mathematics. In the other four categories, two of them are closely related to mathematics (Statistics & Probability and Computer Science, Theory & Methods), while the other two show some efforts in the multidisciplinary and interdisciplinary research. On the other hand, research of Ivan Gutman belongs to 13 categories, and it is far more multidisciplinary and interdisciplinary in its nature. The leading three categories are Chemistry, Multidisciplinary, Mathematics, Applied, and Mathematics, Interdisciplinary Applications. We noticed that Computer Science, Interdisciplinary Applications is the fourth one in the ranking of categories, which is to some extent unexpected, since Gutman is professor of physical chemistry. However, it can be explained by the fact that the journal MATCH: Communications in Mathematical and in Computer Chemistry, where he has published 52 papers, is also enlisted in this WoS category. Taking this into account, a previous study [53] showed that Ivan Gutman is a leading Serbian scientist in all WoS categories related to computer science. However, the results clearly show that the majority of papers written by both scientists belong to applied mathematics, multidisciplinary and interdisciplinary fields.

**Fig. 8:**
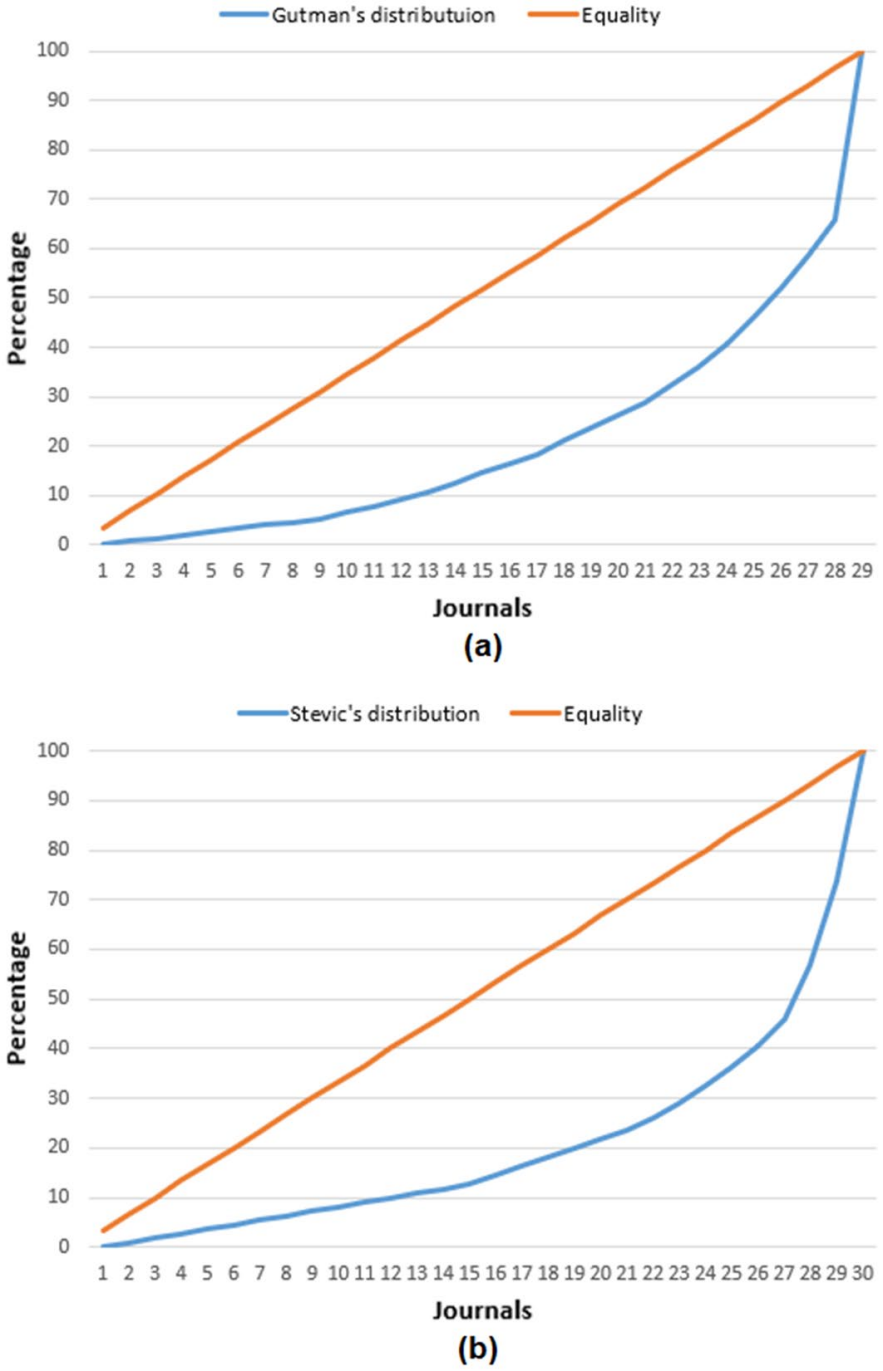
Lorenz curve maps the cumulative papers share on the vertical axis against the number of publishing journals on the horizontal axis; **a** Lorenz curve for Ivan Gutman; **b** Lorenz curve for Stevo Stević

Distribution of papers per journal show significant inequality in favor of two or three journals for both authors. Tables 3 and 4 present journals with more than 5 papers for both authors. Stević published 111 papers in 29 different journals and Gutman published his 152 papers in 28 journals. In order to quantify the inequality, we have calculated Gini coefficient and drew Lorenz curves, using the same methodology described in section International collaboration per country. The Gini coefficient for Stevo Stević is 0.69 and for Ivan Gutman is 0.63. The corresponding Lorenz curves are presented in Fig. 8. Those results correspond to inequality of the number of papers in different journals measured globally, as the value of Gini coefficient 0.65±0.15 [81].

**Table 3:** Number of papers per journal for Ivan Gutman

Journal	No. of papers
Match-Communications in Mathematical and In Computer Chemistry	52
Chemical Physics Letters	11
Polycyclic Aromatic Compounds	10
Linear Algebra and its Applications	9
Journal of the Serbian Chemical Society	8
Journal of Mathematical Chemistry	7
Croatica Chemica Acta	6
Discrete Applied Mathematics	5

**Table 4:** Number of papers per journal for Stevo Stević

Journal	No. of papers
Applied Mathematics and Computation	29
Abstract and Applied Analysis	19
Journal of Difference Equations and Applications	12
Journal of Mathematical Analysis and Applications	6
Journal of Computational Analysis and Applications	5

## Collaboration network analysis

**Fig. 9:**
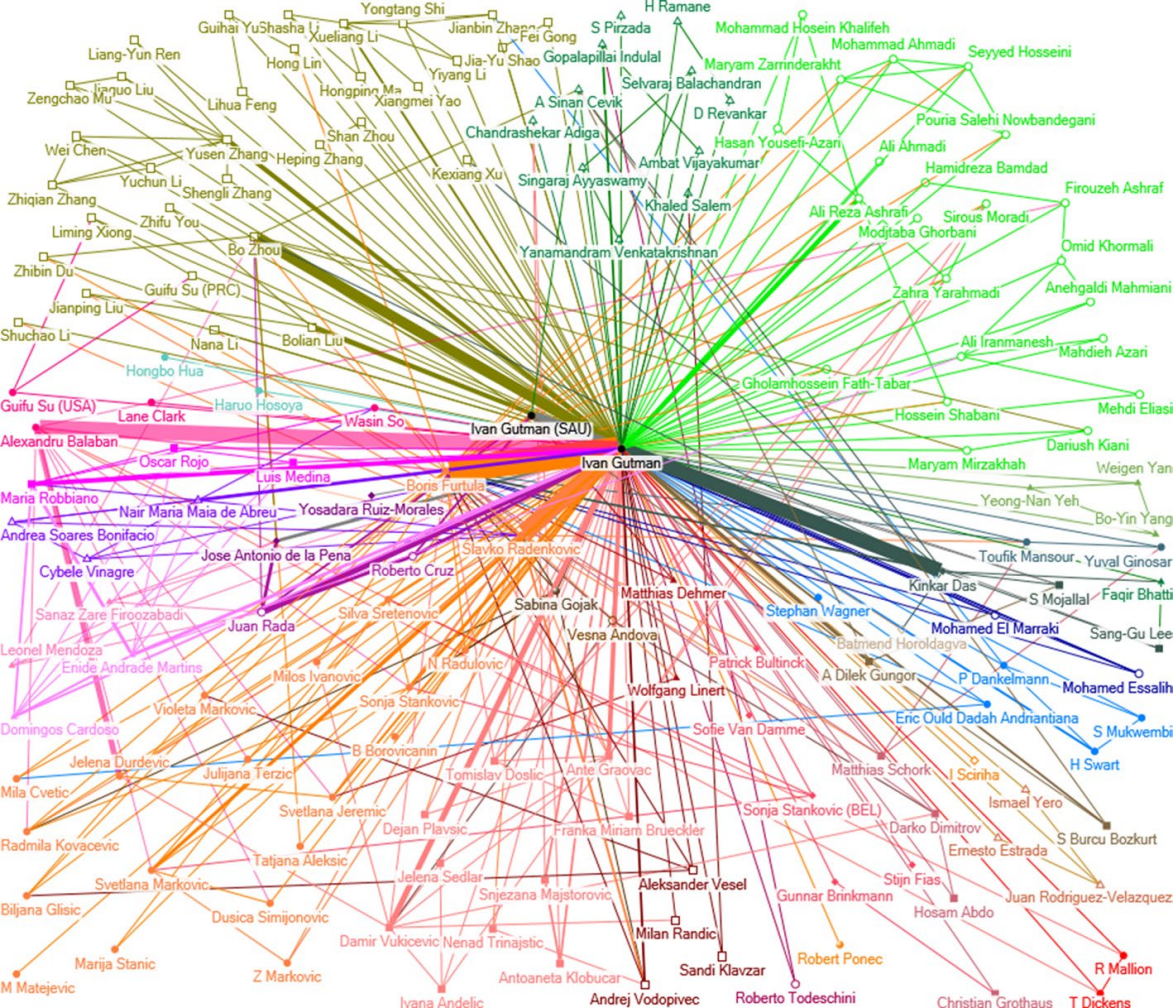
Collaboration network for Ivan Gutman

**Fig. 10:**
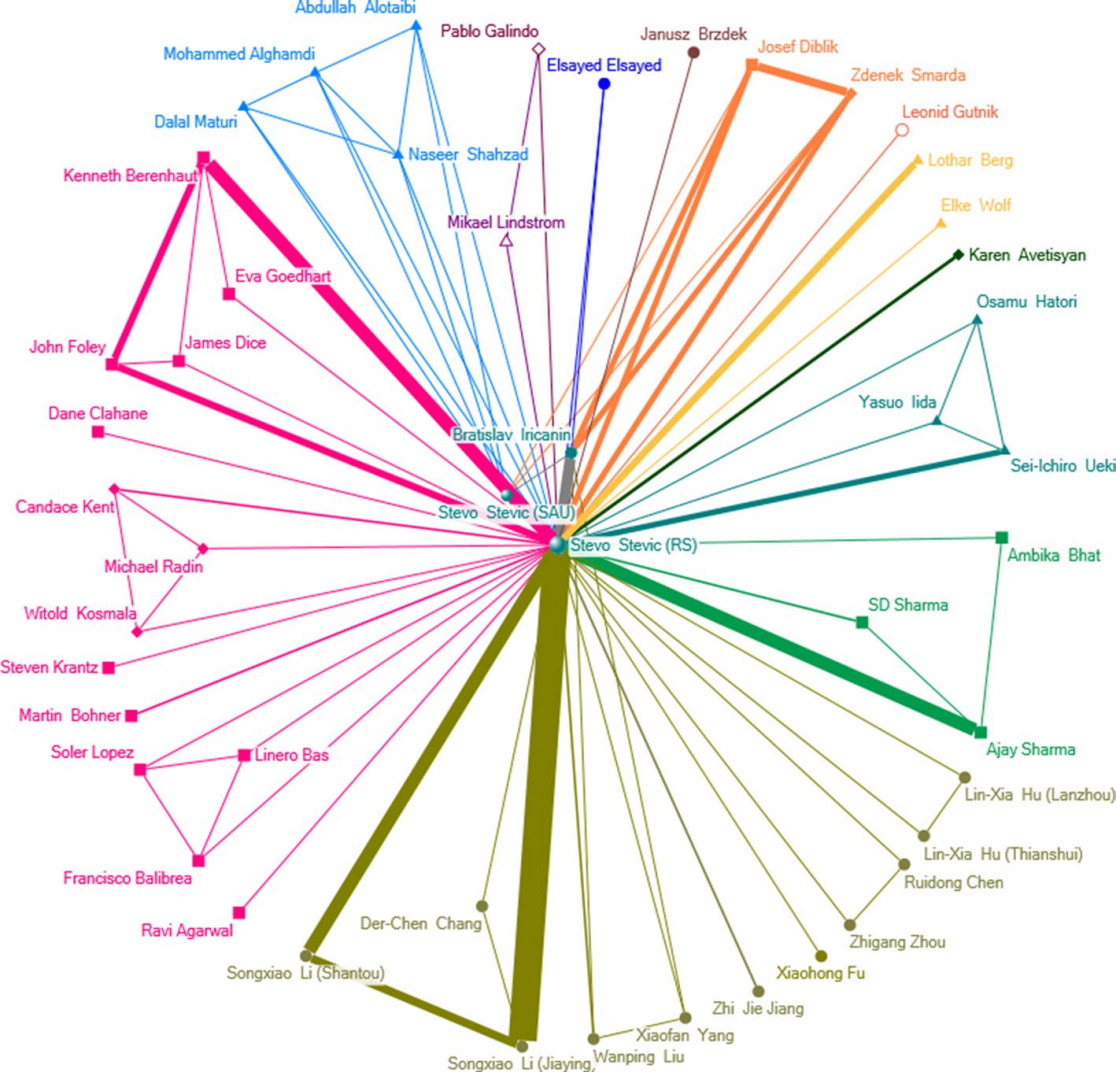
Collaboration network for Stevo Stević

Collaboration networks of Ivan Gutman and Stevo Stević have been analyzed using ego network analysis tools in UCINET. Basic measures are shown in Table 5. Collaboration network of Ivan Gutman and Stevo Stević are shown in Figs. 9 and 10.

**Table 5:** Basic measures for Ivan Gutman and Stevo Stević

Ego	N	Ts	Ps	ND	W	nWC	B	nB	BC	nBC
Ivan Gutman	160	480	25440	1.89	28	17.50	12480.00	0.98	11900.15	93.55
Stevo Stević	47	78	2162	3.61	21	44.68	1042	0.96	1027.75	95.07

NS: network size; Ts: ties; Ps: pairs; ND: network density; WC: number of weak components; nWC: number of weak components divided by size; B: brokerage; NB: normalized brokerage; BC: ego betweenness centrality; nBC: normalized ego betweenness centrality

As it can be observed, both collaboration networks are sparse, as number of ties are much smaller than number of potential pairs. Weak component analysis showed 28 components for Gutman’s collaboration network, and 21 for Stević’s network. In ego network analysis, actor itself (ego node) is excluded from the analysis. The collaboration network graph for Stevo Stević has a clear star shaped structure, as it is indicated by the high BC score of the ego itself [70]. His co-authors are almost always from the same institution from one country. Except Bratislav Iricanin, he did not collaborate with any other Serbian author in his international papers. If we normalize the number of weak components by size, we can observe the importance of the ego node - Stevo Stević is far more important in his network than Ivan Gutman. On the other hand, Ivan Gutman collaborations are far more international: he wrote numerous papers with co-authors from different countries and institutions. Also, numerous authors, both from Serbia and former Yugoslavia, are involved in his international collaboration.

Betweenness centrality analysis shows high scores for both Gutman and Stević, as both actors are highly central in their networks. In Stević’s network, only 8 alters have BC measure higher than 0, including Stević himself with his double affiliation from Saudi Arabia. After Stević, the highest ranked is his co-author from Serbia, Bratislav Iricanin, with normalized betweenness of 26.19. In Gutman’s network, 44 alters have positive betweenness, while top-5 collaborators have betweenness centrality higher than 30. Table 6 shows betweenness centrality measures for top-5 collaborators for both scientists.

**Table 6:** Betweenness centrality measures for top-5 collaborators of Gutman and Stević

Collaborator	Ivan Gutman		Collaborator	Stevo Stević	
	BC	nBC		BC	nBC
Boris Furtula	285.67	42.89	Bratislav Iricanin	5.5	26.19
Slavko Radenkovic	117.9	39.3	Stevo Stevic (SAU)	6.25	22.32
Alexandru Balaban	24.67	31.62	Ajay Sharma	0.5	16.67
Bo Zhou	20.58	31.19	Kenneth Berenhaut	1	16.67
Kinkar Das	13.75	30.56	Songxiao Li (Jiaying)	0.5	16.67

BC: ego betweenness centrality; nBC: normalized ego betweenness centrality

Table 6 provides evidence that Gutman’s co-authors are much more connected than co-authors of Stević, as they play more important, central roles in his collaboration network. To further examine how the collaborators of Ivan Gutman and Stevo Stević are embedded in their neighborhood in analyzed collaboration networks, we have performed the analysis of brokerage roles using UCINET tool. Brokerage roles are based on the studies presented in [70, 82]. Five brokerage roles are defined in [70]: coordinator, consultant, gatekeeper, representative, and liaison. Coordinator connects two members of the same group and belongs to the group. Consultant does not belong to the group but brokers a relation between two members of the same group. Gatekeeper is a member of a group who is at its boundary, and controls access of outsiders to the group. The role of the representative is to act as point of contact between members of the two groups. Representative belongs to one of the groups. On the contrary, liaison connects two groups, but is not part of either. In our collaboration networks, which are represented as undirected graphs, gatekeeper and representative roles are essentially the same.

In both networks, we added attributes to identify originating countries of each collaborator, and then we performed the analysis. The results are shown in Tables 7 and 8. We have taken the top-7 collaborators, since the analysis for Stevo Stević did not show any other brokerage roles among other collaborators. The analysis has found an interesting difference in collaboration patterns of the two scientists that is obviously visible from Figs. 9 and 10. In Stević’s network, only few collaborators have identified brokerage roles of low intensity. The only notable exception is Bratislav Iricanin with the strongest liaison role. This result corresponds to the analysis of betweenness centrality described above. Obviously, Stević does not connect groups from different countries in joint research, but works on individual basis or in small groups from one country.

**Table 7:** Brokerage roles measures for top-7 collaborators of Ivan Gutman

Collaborator	Coordinator	Gatekeeper / Representative	Consultant	Liaison
Boris Furtula	38	190	64	692
Slavko Radenkovic	32	105	14	242
Alexandru Balaban	0	0	50	58
Ivan Gutman (SAU)	0	18	2	66
Bo Zhou	0	9	2	68
Ante Graovac	22	19	0	4
Kinkar Das	2	12	4	28

**Table 8:** Brokerage roles measures for top-7 collaborators of Stevo Stević

Collaborator	Coordinator	Gatekeeper / Representative	Consultant	Liaison
Stevo Stevic (SAU)	2	12	0	0
Bratislav Iricanin	0	0	0	22
Kenneth Berenhaut	4	0	0	0
Mohammed Alghamdi	2	0	0	0
Naseer Shahzad	2	0	0	0
Ajay Sharma	2	0	0	0
Songxiao Li (Jiaying)	2	0	0	0

On the other hand, the collaboration patterns of Ivan Gutman are much more diverse, since his collaborators have different identified roles. The role of the liaison seems to be predominant for his top-5 collaborators. The second most important role is that of the gatekeeper/representative. The diversity of roles shows that professor Gutman is involved in multilateral international cooperation, which results in published papers with coauthors from different countries (groups). Previous analysis showed that professor Gutman cooperated with almost three times more countries than Stević, and has a higher number of coauthors per paper. Those factors affect the potential for international research collaboration, as described in [21].

## Discussion and comparison with the period 2014–2023

This paper focuses on three collaboration patterns that lead to extraordinary publishing performance. In this section, some additional insight on such a high scientific production is given. We compared the obtained results with the ones from the open literature, as well as with the similar dataset for the later period 2014–2023.

### Mega-authorship pattern

As we identified in our primary dataset, one of the prominent collaboration patterns is defined by mega-authorship papers with numerous authors. The number of those papers is on the increase in the last decade, raising questions about the fulfillment of authorship criteria that can be found in [73]. In the more recent period, COVID-19 pandemic and related research led to increase of such mega-authorship papers [83], as 13% of papers related to COVID-19 related research had 10 or more co-authors.

In the context of our work, we observed a significant increase in the number of mega-authorship papers involving Serbian authors. The number of papers rose from 776 to more than 2000 papers for the period 2014-01-01 to 2023-04-01 which follows the period closely analyzed in our study. Vast majority of the papers still come from CMS and ATLAS groups with more than 700 hundred papers both, but there are also seven other groups with more than ten papers. The highest ranked Serbian author, Milosevic J, has 646 papers. It is noticeable that vast majority of the papers are open access. More than hundred papers are highly cited papers according to WoS statistics.

### Collaboration patterns of individual researchers

According to [43], the best method to analyze individual publishing performance is to combine bibliometrics with qualitative information from the biographies and working context. Our analysis showed that Stević mainly acts as a solitary author, while professor Gutman maintained strong relations with many co-authors. Publishing performance of the two authors showed obvious differences, as presented in the results of the statistical analysis. Ivan Gutman has over three times more identified coauthors, and the average number of authors per paper is 0.8 greater for Gutman than for Stević. The number of collaborating countries is 2.5 times greater for Gutman. The number of WoS categories of journals that published their papers was 13 for Gutman and 6 for Stević. The only notable similarity shown in statistical analysis is inequality expressed by Gini coefficient of around 0.65 for distribution of papers per journal for both authors.

It was interesting to compare international publishing performance of Ivan Gutman and Stevo Stević with the production that includes only Serbian coauthors (domestic papers). In the same period, Stevo Stević has published 149 papers, out of which 143 were single authored papers. The rest six papers were co-authored with one domestic co-author. Stević was corresponding author for all papers. In that sense, we consider Stevo Stević as a solitary author at the national level, which is in accordance with classification in [20]. On the other hand, his collaboration network is almost exclusively international. Almost a half of his domestic papers were published in a single journal, Applied Mathematics and Computation, which is even more noticeable trend than in his international production. Ivan Gutman had 70 domestic papers in the same period, out of which ten papers were single authored, but for 56 papers he was corresponding author. Considering the total number of domestic and international papers, Stevo Stević has even greater productivity in the analyzed period than Ivan Gutman, who has more coauthors in total and greater international collaboration network.

We compared the obtained results with the period after 2013. The total number of papers that satisfied our query increased roughly 2.5 times. After excluding mega-authorship papers, two researchers, Marina Soković (247 papers) and Dragan Pamučar (219 papers) had significantly higher scientific production than others on the list. For that reason, we decided to compare those two researchers with Gutman and Stević, as they seem to be in the comparable position to the period 2006-2013. It should be noted that Gutman and Stević are still high on the list, with 69 and 118 published papers, respectively.

**Fig. 11:**
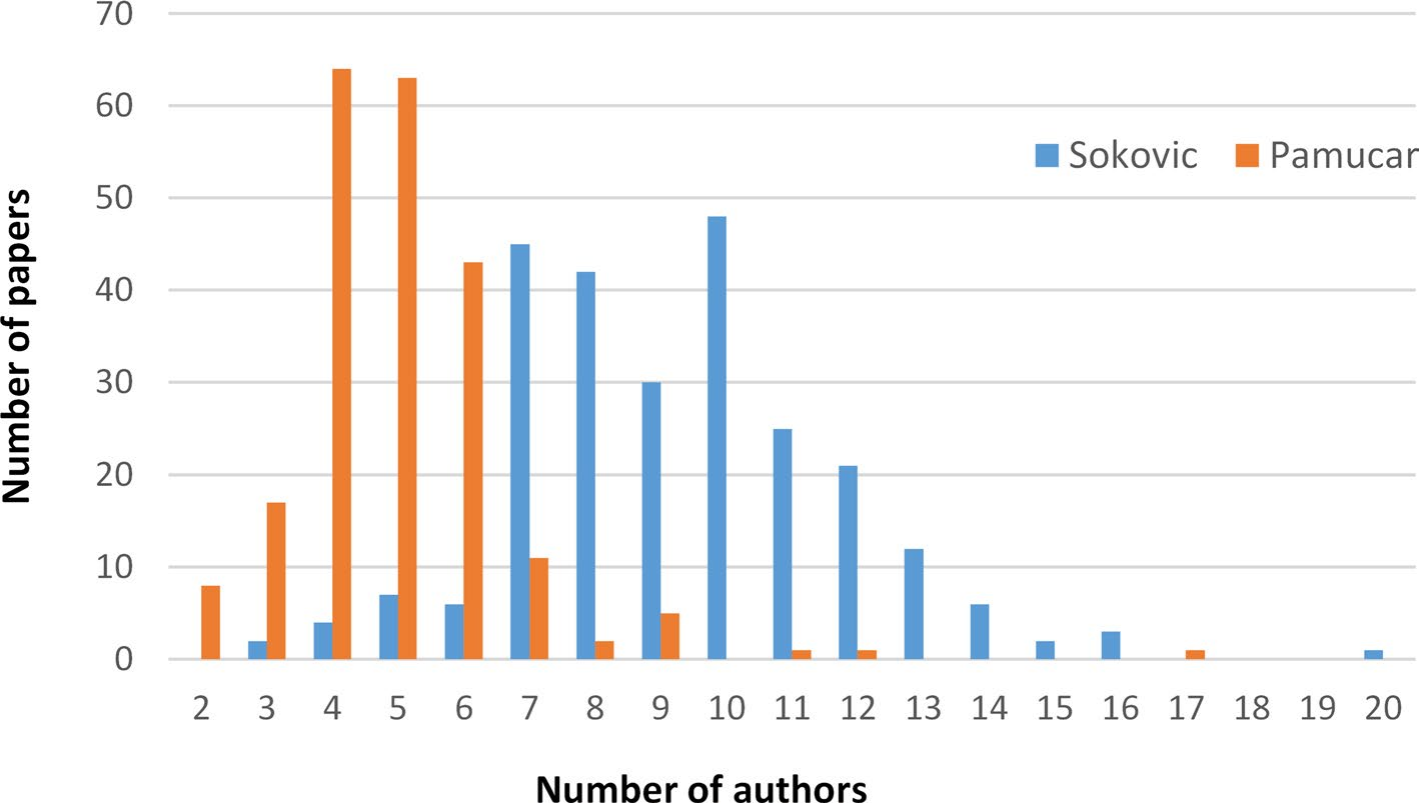
Distribution of papers per number of authors for Marina Soković and Dragan Pamučar

First notable difference from Stević and Gutman is the number of papers per author for those two authors, Soković and Pamučar, that can be seen in Fig. 11. Average number of papers per author for Pamučar is 4.97, while for Soković is 9.29 which is a significant increase over both Stević (2.56) and Gutman (3.36). However, it is in accordance with the findings from the open literature [62] where increased average number of authors per publication found some criticism [84]. Increased number of co-authors is also reflected in the number of countries both Soković and Pamučar cooperate with. Soković collaborates with researchers from 47 countries, while Pamučar collaborates with researchers from 55 countries. In that sense, both researchers are significantly involved in international collaboration. However, the most of the Soković’s collaborations are established with Mediterranean countries, such as Portugal, Greece, Spain, and Italy, and Turkey, as shown in Table 9. Pamučar’s collaborations are widely established with Asian countries, such as Turkey, Pakistan, India, China, and Saudi Arabia.

**Table 9:** Top-5 collaborating countries for Marina Soković and Dragan Pamučar

Marina Soković		Dragan Pamučar	
Country	No. of papers	Country	No. of papers
Portugal	156	Turkey	63
Greece	80	Pakistan	62
Spain	43	India	51
Italy	24	China	30
Turkey	22	Saudi Arabia	25

Marina Soković publishes mostly in the domain of food science technology, biochemistry, molecular biology, agronomy, and plant sciences. Dragan Pamučar is involved in interdisciplinary and multidisciplinary applications in computer science (artificial intelligence), environmental sciences, green sustainable science technology, and operations research. The number of journals with 5 or more papers is also higher than for Gutman and Stević, as shown in Tables 10 and 11. In both cases inequality in distribution of the papers over journals can be observed, similar to Gutman and Stević.

**Table 10:** Number of papers per journal for Marina Soković

Journal	No. of papers
Food Function	32
Molecules	21
Industrial Crops and Products	16
Food Chemistry	14
Antioxidants	9
Food Research International	9
LWT Food Science and Technology	9
Foods	7
Pharmaceuticals	7
Antibiotics Basel	6
Current Topics in Medicinal Chemistry	6
Medchemcomm	5

**Table 11:** Number of papers per journal for Dragan Pamučar

Journal	No. of papers
Symmetry Basel	30
Mathematics	13
Expert Systems with Applications	11
Computational Intelligence and Neuroscience	9
Mathematical Problems in Engineering	9
Sustainability	7
Entropy	6
International Journal of Intelligent Systems	6
Journal of Cleaner Production	6
Applied Soft Computing	5
Axioms	5
Discrete Dynamics in Nature And Society	5

To put our study in the global and regional context, we compared all four researchers according to the Stanford list based on career-long statistic for 2021 compiled by Ioannidis et al. [85, 86] based on Scopus data. Each researcher is classified into one out of 22 scientific fields, as well as into two out of 176 subfields. We can observe from Table 12 that Gutman and Stević are highly ranked in their common subfield of General Mathematics globally. Their international scientific impact can be also observed in WoS where Gutman has 7, and Stević has 8 highly cited papers. Soković and Pamučar are ranked in %1.5 percent of the top-ranked authors in their respective subfields. However, it should be noted that the subfield of Pamučar has an order of magnitude more researchers ranked in this dataset. Their international scientific impact is also significant, as Soković has 3, and Pamučar has 17 highly cited papers in WoS.

In the regional context of the six former Yugoslavia republics, there are 314 researchers on the Stanford career-long statistic list for 2021. There are 10 mathematicians on the list where Gutman and Stević are obviously on the top of the list, both based on the h-index and their corresponding subfield ranks. Soković is ranked first among researchers from former Yugoslavia republics based on h-index for 2021. In his subfield of Artificial Intelligence & Image Processing, Pamučar is ranked fourth based on his h-index for 2021, after three researchers from Slovenia.

**Table 12:** Ranking of four authors based on Scopus data [85, 86]

Author	YFP	H21	F	SF	WR
Ivan Gutman	1972	70	Mathematics & Statistics	General Mathematics	7/80129
Stevo Stević	2001	67	Mathematics & Statistics	General Mathematics	8/80129
Marina Soković	1999	46	Chemistry	Medicinal & Biomolecular Chemistry	706/99546
Dragan Pamučar	2011	35	Information & Communication Technologies	Artificial Intelligence & Image Processing	8564/546033

YFP: year of first publication; H21: h-index as of end-2021; F: scientific field; SF: top-ranked subfield; WR: world rank in the subfield/all ranked in the subfield

We can say that for all analyzed researchers international cooperation played key role in their successful careers. Similar conclusions were made in [87] where author analyzed international and local cooperation of Polish authors. Also, our analysis showed that multidisciplinary, interdisciplinary, and applied sciences represent a promising polygon for high scientific production. Multidisciplinary approaches to mathematics with applications in other scientific fields (chemistry, physics, life sciences, computer sciences) are fruitful for extensive scientific production. It is also shown that those fields attract interest for joint cooperation, as is the case with Australia and South Korea [35].

This trend is noticeable in the case of Ivan Gutman, since his research interests are mainly focused on the graph theory and its application in chemistry, which makes his research multidisciplinary. In the subsequent years, Dragan Pamučar tends to follow similar path. Earlier studies, such as [88], also showed that researchers in the physical sciences and mathematics are more actively involved in international collaboration.

On the other hand, the publishing performances of Marina Soković are in the accordance with the constantly high ranking of the University of Belgrade in the ARWU list in the domain of Food Science and Technology, where in the year 2022 it was ranked between 51 and 75 places. Her research fields also span to the fields of applied chemistry, pharmacy, and pharmacology, which are the good foundations for high scientific production. Stevo Stević is the most unique of the analyzed researchers in the sense that his joint work is mostly conducted with relatively small groups of researchers from single country. He cooperates more in bilateral fashion, which is earlier observed in the studies of Mexican researchers both from the domain of social sciences [41] and chemistry [40].

## Conclusion

Nowadays, scientometrics research includes dealing with the large quantity of data, which are deeply processed to produce meaningful results at the individual level. In our research, we have identified three interesting collaboration patterns which lead to high productivity of scientific papers with international co-authorship. We studied international scientific collaboration patterns using dataset that included research papers co-authored by at least one author from Serbia and at least one author from abroad. We performed detailed analysis for the the period 2006–2013, and gave addiotional insights for the period 2014–2023. First collaboration pattern corresponds to mega-authorship papers with hundreds of co-authors gathered in institutional research groups. The other two collaboration patterns were found in mathematics and multidisciplinary science, mainly application of graph theory and computational methods in physical chemistry which we examined in more details on the example of two most productive scientists in the dataset.

There are several directions for future work. In order to analyze the scientific impact of their research, we plan to perform citation analysis of the prominent authors, together with impact factor analysis of the journals of their publications. Also, we plan to expand collaboration networks of prominent scientists and analyze their two-step neighborhoods. The analysis presented in this paper will be followed by an exhaustive investigation of overall international collaboration of all Serbian researchers, considering scientific fields and collaborating countries.

The global increase of overall scientific production, as well as the number of authors per paper, citations, and self-citations, during the last two decades, also raised some questions about anomalies that should be explored by the scientific community. In the future, we plan to examine and propose some compound metrics to evaluate those issues.

## Data Availability

The datasets used and/or analyzed during the current study are available from the corresponding author on reasonable request.

## Abbreviations

GDP: Gross Domestic Product
R&D: Research and Development
WoS: Web of Science

## References

[1] Archambault É, Campbell D, Gingras Y, Larivière V (2009). Comparing bibliometric statistics obtained from the Web of Science and Scopus. J Am Soc Inf Sci Technol..

[2] Swan A (2007). Macroscope: Open access and the progress of science. Am Sci..

[3] OST-Science, Observatory T. Dynamics of scientific production in the world, in Europe and in France, 2000-2016. Hcéres París; 2019.

[4] Bornmann L, Mutz R (2015). Growth rates of modern science: a bibliometric analysis based on the number of publications and cited references. J Assoc Inf Sci Technol..

[5] White K. Publications Output: US Trends and International Comparisons. Science & Engineering Indicators 2022. NSB-2021-4. National Science Board, National Science Foundation; 2021.

[6] Safder I, Hassan SU (2019). Bibliometric-enhanced information retrieval: a novel deep feature engineering approach for algorithm searching from full-text publications. Scientometrics..

[7] Correia A, Jameel S, Schneider D, Paredes H, Fonseca B. A workflow-based methodological framework for hybrid human-AI enabled scientometrics. In: 2020 IEEE International Conference on Big Data (Big Data). IEEE; 2020. p. 2876–2883.

[8] Moral-Muñoz JA, Herrera-Viedma E, Santisteban-Espejo A, Cobo MJ (2020). Software tools for conducting bibliometric analysis in science: an up-to-date review. Profesional de la Información..

[9] Staegemann D, Volk M, Daase C, Turowski K (2020). Discussing relations between dynamic business environments and big data analytics. Complex Syst Inf Model Q..

[10] Melnikova E (2022). Big data technology in the set of methods and means of scientific research in modern scientometrics. Sci Techn Inf Process..

[11] Lopez-Rodriguez V, Ceballos HG (2022). Modeling scientometric indicators using a statistical data ontology. J Big Data..

[12] Marginson S (2022). Global science and national comparisons: beyond bibliometrics and scientometrics. Comp Educ..

[13] Xu HY, Yue ZH, Wang C, Dong K, Pang HS, Han Z (2017). Multi-source data fusion study in scientometrics. Scientometrics..

[14] D’Angelo CA, van Eck NJ (2020). Collecting large-scale publication data at the level of individual researchers: a practical proposal for author name disambiguation. Scientometrics..

[15] Price DJ (1986). Little science, big science... and beyond.

[16] Larsen P, Von Ins M (2010). The rate of growth in scientific publication and the decline in coverage provided by Science Citation Index. Scientometrics..

[17] Ivanović D, Ho YS (2014). Independent publications from Serbia in the Science Citation Index Expanded: a bibliometric analysis. Scientometrics..

[18] Ivanović D, Fu HZ, Ho YS (2015). Publications from Serbia in the Science Citation Index Expanded: a bibliometric analysis. Scientometrics..

[19] Adams J (2012). The rise of research networks. Nature..

[20] Ioannidis JP (2008). Measuring co-authorship and networking-adjusted scientific impact. PLoS ONE..

[21] Haddow G. International Research Collaboration: A Working Model. In: IFLA 2013 Satellite Meeting: Workshop on Global Collaboration of Information Schools. Nanyang Technology University; 2013. p. 11–18.

[22] Buela-Casal G, Perakakis P, Taylor M, Checa P (2006). Measuring internationality: reflections and perspectives on academic journals. Scientometrics..

[23] Chen K, Zhang Y, Fu X (2019). International research collaboration: an emerging domain of innovation studies?. Res Policy..

[24] Moed HF, de Moya-Anegon F, Guerrero-Bote V, Lopez-Illescas C (2020). Are nationally oriented journals indexed in Scopus becoming more international? The effect of publication language and access modality. J Informetrics..

[25] Gazni A, Sugimoto CR, Didegah F (2012). Mapping world scientific collaboration: authors, institutions, and countries. J Am Soc Inf Sci Technol..

[26] Havemann F, Heinz M, Kretschmer H (2006). Collaboration and distances between German immunological institutes-a trend analysis. J Biomed Discov Collab..

[27] Waltman L, Tijssen RJ, van Eck NJ (2011). Globalisation of science in kilometres. J Informetr..

[28] Csomós G (2018). A spatial scientometric analysis of the publication output of cities worldwide. J Informetr..

[29] Aman V (2018). Does the Scopus author ID suffice to track scientific international mobility? A case study based on Leibniz laureates. Scientometrics..

[30] Ortega JL, Aguillo IF (2013). Institutional and country collaboration in an online service of scientific profiles: Google Scholar Citations. J Informetr..

[31] Martín-Martín A, Thelwall M, Orduna-Malea E, Delgado López-Cózar E (2021). Google Scholar, Microsoft Academic, Scopus, Dimensions, Web of Science, and OpenCitations’ COCI: a multidisciplinary comparison of coverage via citations. Scientometrics..

[32] Glänzel W, Schubert A, Czerwon HJ (1999). A bibliometric analysis of international scientific cooperation of the European Union (1985–1995). Scientometrics..

[33] Teodorescu D, Andrei T (2011). The growth of international collaboration in East European scholarly communities: a bibliometric analysis of journal articles published between 1989 and 2009. Scientometrics..

[34] Onyancha OB, Maluleka JR (2011). Knowledge production through collaborative research in sub-Saharan Africa: how much do countries contribute to each other’s knowledge output and citation impact?. Scientometrics..

[35] Choi M, Lee H, Zoo H (2021). Scientific knowledge production and research collaboration between Australia and South Korea: patterns and dynamics based on co-authorship. Scientometrics..

[36] Huang MH, Tang MC, Chen DZ (2011). Inequality of publishing performance and international collaboration in physics. J Am Soc Inf Sci Technol..

[37] Prathap G (2017). Scientific wealth and inequality within nations. Scientometrics..

[38] Vanni T, Mesa-Frias M, Sanchez-Garcia R, Roesler R, Schwartsmann G, Goldani MZ (2014). International Scientific Collaboration in HIV and HPV: a network analysis. PLoS ONE.

[39] Wu Y, Duan Z (2015). Social network analysis of international scientific collaboration on psychiatry research. Int J Mental Health Syst..

[40] Russell JM, Hernández-García Y, Kleiche-Dray M (2016). Collaboration dynamics of Mexican research in Chemistry and its relationship with communication patterns. Scientometrics..

[41] González Brambila CN, Olivares-Vázquez JL (2021). Patterns and evolution of publication and co-authorship in Social Sciences in Mexico. Scientometrics..

[42] Fu YC, Marques M, Tseng YH, Powell JJ, Baker DP (2022). An evolving international research collaboration network: spatial and thematic developments in co-authored higher education research, 1998–2018. Scientometrics..

[43] Glänzel W (2014). Analysis of co-authorship patterns at the individual level. Transinformação..

[44] Uddin S, Hossain L, Rasmussen K (2013). Network effects on Scientific Collaborations. PLoS ONE.

[45] Newman ME (2004). Coauthorship networks and patterns of scientific collaboration. Proc Natl Acad Sci..

[46] Isfandyari-Moghaddam A, Saberi MK, Tahmasebi-Limoni S, Mohammadian S, Naderbeigi F. Global scientific collaboration: A social network analysis and data mining of the co-authorship networks. Journal of Information Science. 2021;p. 01655515211040655.

[47] Hirsch JE (2010). An index to quantify an individual’s scientific research output that takes into account the effect of multiple coauthorship. Scientometrics..

[48] Ausloos M (2013). A scientometrics law about co-authors and their ranking: the co-author core. Scientometrics..

[49] Ivanović D, Jovanović M, Fritsche F (2016). Analysis of scientific productivity and cooperation in the republics of former Yugoslavia before, during and after the Yugoslav wars. Scientometrics..

[50] Uskoković V, Ševkušić M, Uskoković D. Strategies for the Scientific Progress of the Developing Countries in the New Millennium: The cases of Serbia, Slovenia and South Korea. Science, Technology & Innovation Studies. 2010;p. 33–62.

[51] Popovic A, Antonic S. Statistical analysis of citation results for researchers in Serbia. In: INFORUM 2011: 17th Conference on Professional Information Resources; 2010.

[52] Savić M, Ivanović M, Radovanović M, Ognjanović Z, Pejović A, Jakšić Krüger T (2014). The structure and evolution of scientific collaboration in Serbian mathematical journals. Scientometrics..

[53] Pavkovi ć M, Protić J. An analysis of scientific publications from Serbia: The case of computer science. Math Comput Model. 2015;47:2–020.

[54] Dong K, Wu J, Wang K (2021). On the inequality of citation counts of all publications of individual authors. J Informetr..

[55] Abramo G, Cicero T, D’Angelo CA (2013). Individual research performance: a proposal for comparing apples to oranges. J Informetr.

[56] Fischer D, Nordhausen K, Taskinen S. Publication and Coauthorship Networks of Hannu Oja. In: Modern Nonparametric, Robust and Multivariate Methods. Springer; 2015. p. 7–27.

[57] Lu W, Ren Y, Huang Y, Bu Y, Zhang Y (2021). Scientific collaboration and career stages: an ego-centric perspective. J Informetr.

[58] Arnaboldi V, Dunbar RI, Passarella A, Conti M. Analysis of co-authorship ego networks. In: International Conference and School on Network Science. Springer; 2016. p. 82–96.

[59] Díaz-Faes AA, Llopis O, D’Este P, Molas-Gallart J. Assessing the variety of collaborative practices in translational research: An analysis of scientists’ ego-networks. Research Evaluation. 2023;p. rvad003.

[60] Kong X, Mao M, Jiang H, Yu S, Wan L (2019). How does collaboration affect researchers’ positions in co-authorship networks?. J Informetr..

[61] Academic Ranking of World Universities; 2013. Accessed: 2022-07-12. http://www.shanghairanking.com/.

[62] Glänzel W, Schubert A. Analysing scientific networks through co-authorship. In: Handbook of quantitative science and technology research. Springer; 2004. p. 257–276.

[63] Smalheiser NR, Torvik VI (2009). Author name disambiguation. Ann Rev Inf Sci Technol..

[64] Tang L, Walsh J (2010). Bibliometric fingerprints: name disambiguation based on approximate structure equivalence of cognitive maps. Scientometrics..

[65] Torvik VI, Weeber M, Swanson DR, Smalheiser NR (2005). A probabilistic similarity metric for Medline records: a model for author name disambiguation. J Am Soc Inf Sci Technol.

[66] Milojević S (2013). Accuracy of simple, initials-based methods for author name disambiguation. J Informetr.

[67] Hussain I, Asghar S (2017). A survey of author name disambiguation techniques: 2010–2016. Knowl Eng Rev..

[68] Shoaib M, Daud A, Amjad T. Author name disambiguation in bibliographic databases: A survey. arXiv preprint arXiv:2004.06391. 2020;.

[69] Mitrovic I, Protic J. Problems with affiliations, names and personal identity in the proces of evaluating higher education institutions. In: 6th International Conference on Education and New Learning Technologies; 2014.

[70] Hanneman RA, Riddle M (2005). Introduction to social network methods.

[71] Smith MA, Shneiderman B, Milic-Frayling N, Mendes Rodrigues E, Barash V, Dunne C, et al. Analyzing (social media) networks with NodeXL. In: Proceedings of the fourth international conference on Communities and technologies; 2009. p. 255–264.

[72] Borgatti SP, Everett MG, Freeman LC (2002). Ucinet for Windows: Software for social network analysis. Harvard, MA: analytic technologies..

[73] Byard RW, Vink R. Does listing of individual contributions in “mega-authorship” papers always follow best practice guidelines? Springer; 2021. 10.1007/s12024-021-00388-8 34061316

[74] Sriram P (2015). India: Multi-author papers skew ranking. Nature..

[75] Stojiljkovic S. Srpski dvojac blistavih umova; 2013. Accessed: 2022-07-12. http://www.politika.rs/scc/clanak/274968/Srpski-dvojac-blistavih-umova.

[76] Home page - Ivan Gutman; 2021. Accessed: 2022-07-12. http://www.pmf.kg.ac.rs/gutman/.

[77] Watch CAS. Rising stars; 2011. Accessed: 2022-08-07. http://archive.sciencewatch.com/dr/rs/.

[78] Lindsey D (1980). Production and citation measures in the sociology of science: the problem of multiple authorship. Soc Stud Sci..

[79] Xu J, Ding Y, Song M, Chambers T (2016). Author credit-assignment schemas: a comparison and analysis. J Assoc Inf Sci Technol..

[80] Ho YS (2013). The top-cited research works in the Science Citation Index Expanded. Scientometrics..

[81] Ghosh A, Chattopadhyay N, Chakrabarti BK (2014). Inequality in societies, academic institutions and science journals: Gini and k-indices. Physica A: Stat Mech Appl..

[82] Gould RV. Fernandez RM. Structures of mediation: a formal approach to brokerage in transaction networks. Sociological methodology; 1989. p. 89–126.

[83] Pal JK (2021). Visualizing the knowledge outburst in global research on COVID-19. Scientometrics..

[84] Hosseini M, Lewis J, Zwart H, Gordijn B (2022). An ethical exploration of increased average number of authors per publication. Sci Eng Ethics..

[85] Ioannidis JP, Baas J, Klavans R, Boyack KW (2019). A standardized citation metrics author database annotated for scientific field. PLoS Biol..

[86] Ioannidis JP, Boyack KW, Baas J (2020). Updated science-wide author databases of standardized citation indicators. PLoS Biol..

[87] Kwiek M (2020). Internationalists and locals: international research collaboration in a resource-poor system. Scientometrics..

[88] Piro FN, Aksnes DW, Rørstad K (2013). A macro analysis of productivity differences across fields: challenges in the measurement of scientific publishing. J Am Soc Inf Sci Technol..

